# Estimates of mortality attributable to COVID-19: a statistical model for monitoring COVID-19 and seasonal influenza, Denmark, spring 2020

**DOI:** 10.2807/1560-7917.ES.2021.26.8.2001646

**Published:** 2021-02-25

**Authors:** Jens Nielsen, Naja Hulvej Rod, Lasse S Vestergaard, Theis Lange

**Affiliations:** 1 Infectious Disease Epidemiology and Prevention, Statens Serum Institut, Denmark; 2 Section of Epidemiology, Department of Public Health, University of Copenhagen, Denmark; 3 Section of Biostatistics, Department of Public Health, University of Copenhagen, Denmark

**Keywords:** Mortality, Surveillance, Influenza, COVID-19

## Abstract

**Background:**

Timely monitoring of COVID-19 impact on mortality is critical for rapid risk assessment and public health action.

**Aim:**

Building upon well-established models to estimate influenza-related mortality, we propose a new statistical Attributable Mortality Model (AttMOMO), which estimates mortality attributable to one or more pathogens simultaneously (e.g. SARS-CoV-2 and seasonal influenza viruses), while adjusting for seasonality and excess temperatures.

**Methods:**

Data from Nationwide Danish registers from 2014-week(W)W27 to 2020-W22 were used to exemplify utilities of the model, and to estimate COVID-19 and influenza attributable mortality from 2019-W40 to 2020-W20.

**Results:**

SARS-CoV-2 was registered in Denmark from 2020-W09. Mortality attributable to COVID-19 in Denmark increased steeply, and peaked in 2020-W14. As preventive measures and national lockdown were implemented from 2020-W12, the attributable mortality started declining within a few weeks. Mortality attributable to COVID-19 from 2020-W09 to 2020-W20 was estimated to 16.2 (95% confidence interval (CI): 12.0 to 20.4) per 100,000 person-years. The 2019/20 influenza season was mild with few deaths attributable to influenza, 3.2 (95% CI: 1.1 to 5.4) per 100,000 person-years.

**Conclusion:**

AttMOMO estimates mortality attributable to several pathogens simultaneously, providing a fuller picture of mortality by COVID-19 during the pandemic in the context of other seasonal diseases and mortality patterns. Using Danish data, we show that the model accurately estimates mortality attributable to COVID-19 and influenza, respectively. We propose using standardised indicators for pathogen circulation in the population, to make estimates comparable between countries and applicable for timely monitoring.

## Introduction

The corona virus disease (COVID-19) pandemic, caused by the severe acute respiratory syndrome coronavirus 2 (SARS-CoV-2) has, as up to 31 October 2020, resulted in 45.4 million confirmed infections and 1.2 million deaths globally [1]. Non-pharmaceutical interventions have been implemented worldwide and many countries have been under strict lockdown. To provide effective pandemic risk assessment, preparedness and response, including evaluation of the effect of implemented measures in society, timely monitoring of COVID-19-attributable outcomes is critical.

Mortality is a basic indicator of population health, and mortality surveillance is fundamental for effective public health planning and action. COVID-19 case fatality has been one of the indicators most referred to worldwide during the pandemic, but this measure entails a number of limitations. In general, deaths from COVID-19 are expected to be undercounted due to not enough testing or testing capacity and due to false-negative test results in severely ill patients [2]. Further, COVID-19 fatality is often counted as case fatality, i.e. number of all-cause deaths within a fixed period after a positive SARS-CoV-2 test (normally 30 days). Thus, deaths that are caused by COVID-19 but occur after 30 days of a positive test will not be counted, and deaths occurring prior to 30 days after the test, but unrelated to COVID-19, will be misclassified as COVID-19 deaths.

An alternative to COVID-19 case fatality is to expand well-established models developed to estimate excess mortality related to seasonal influenza. This approach should provide estimates of excess mortality including deaths both directly and indirectly related to COVID-19, while taking into account underlying patterns in mortality, extreme temperatures and seasonal influenza.

A commonly used methodology for estimating excess mortality is modelling expected mortality based on predefined periods with no or ignorable influenza activity. This baseline, which usually is fitted with a cyclic variation over the year (seasonality) [3], can be compared with the observed number of deaths, and the difference represents an estimate of the excess mortality. Since 2009, the network for European monitoring of excess mortality for public health action (EuroMOMO) has monitored weekly excess all-cause mortality [4,5] using this method. The estimated excess mortality has attracted high attention during the COVID-19 pandemic, and has been used as a proxy for timely monitoring of mortality related to COVID-19. Using and expanding this existing infrastructure to also cover timely monitoring of mortality attributable to COVID-19 could form a valuable alternative or supplement to the COVID-19 case fatality.

Within the EuroMOMO framework, the FluMOMO model [6] was designed to estimate mortality attributable to influenza based on influenza activity, adjusting for extreme ambient temperatures. With the COVID-19 pandemic, there is a need to expand this model to also include COVID-19. We have extended and improved this model into a general Attributable Mortality Monitoring (AttMOMO) model, enabling estimation of attributable mortality from several pathogens simultaneously, e.g. COVID-19 and seasonal influenza. We applied the model on high-quality data from nationwide Danish Health Registers and estimated COVID-19 and influenza-attributable mortality in Denmark from October 2019 to May 2020.

## Methods

### The Attributable Mortality Monitoring (AttMOMO) model

For timely surveillance of mortality due to for example COVID-19, we propose a general model decomposing the total number of deaths into those attributable to one or more infectious pathogens circulating in a population, and those attributable to deaths due to excess temperatures and other seasonal patterns. This model will be applicable in situations with several infectious pathogens circulating simultaneously, such as influenza and COVID-19, and can also be used for simultaneous estimation of for example influenza and respiratory syncytial virus (RSV), or for mortality attributable to various subtypes of influenza.

Number of deaths in a population may be decomposed as:

deaths = Σ_i_ (deaths attributable to *pathogen_i_*) + deaths due to excess temperatures + deaths due to other causes.

This may be written as:

deaths = *Σ_i_ icf_i_* × *(*infected by* pathogen_i_)* + deaths due to excess temperatures + deaths due to other causes,

where *pathogen_i_*is a given pathogen i, and *icf_i_*is the infection-case-fatality for *pathogen_i_*, adjusted for other pathogens included in the model, as well as deaths due to excess temperatures and other causes. The expression ‘infected by* pathogen_i_*‘ is the number of persons infected with *pathogen_i_* in the population.

For inference, an additive, multivariable, time series regression with the following mean structure for the death count can be applied:

$$E\left(D_{t}\right)\;=\;\Sigma_{i}\left(\Sigma_{s}\beta_{1,i,s}\times PA_{t,i,s}\right)\;+\;\Sigma_{p}\beta_{2,t,p}\times ET_{t,p}\;+\;\;{\text{baseline}}_{t}$$ (1)

where *D* is observed number of deaths, *β* are regression coefficients and *p* represents four excess temperature parameters. The time* t* is in weeks and *s* is the season defined as week 27 to week 26 the following year.

Baseline, representing ‘deaths due to other causes’, consists of an overall level (intercept), a linear trend and seasonality.

Excess temperatures, *ET*, i.e. temperatures outside an expected normal range for a week, are included as four continuous parameters, representing excess cold in the winter (may be associated with increased mortality) and excess cold in the summer (may be associated with lower mortality). Conversely, periods with excess heat may be associated with increased mortality in summer, but lower mortality in winter.

For a pathogen, *i*, *PA_t,i,s_* is the number of infected persons, in week *t* and season *s*. A separate effect for each pathogen in each season is included, as the number of infected persons by a pathogen may vary between seasons.

Deaths attributable to a given pathogen will most likely happen with a delay. Hence, in the full model, time-lagged effects of the pathogens as well as excess temperature are included.

The number of attributable deaths due to each pathogen can be achieved using a conditional approach i.e. what would the estimated effect of one pathogen be, had there been no excess temperature and no other pathogens were circulating. This will be an estimation of deviation from the baseline, referred to onward as the ‘base model’.

**Figure 1:**
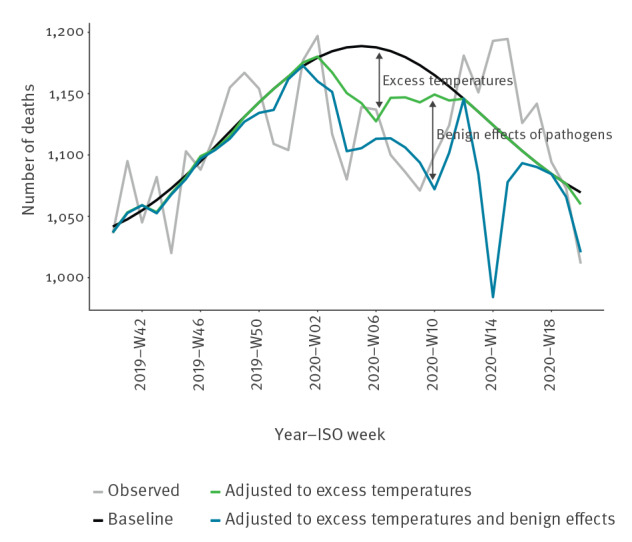
Distribution over time of observed number of deaths, as well as baseline deathsand adjusted baseline deaths, Denmark, 2019-W40–2020-W20

ISO: International Organization for Standardization; W: week.

Awareness of circulation of a pathogen may promote protective measures such as social distancing practiced during the COVID-19-pandemic. Such interventions will also reduce the risk of infection by other pathogens. Hence, the estimated effect of one pathogen may be blurred by indirect benign/lifesaving effects from other pathogens. Further, if a pathogen has been circulating for more than one season, there may be seasons with low circulation or in which the pathogen is less infectious; for influenza virus for example, this would be a ‘mild’ influenza season. The model may for that season estimate benign effects, i.e. death counts below the baseline. Therefore, the effect of one pathogen should also be adjusted for benign effects of other pathogens and excess temperatures, i.e. as deviation from a baseline adjusted to benign effects (Figure 1), referred to onward as the ‘adjusted baseline’ model.

$$E\left(D_{t}\right)\;=\;\Sigma_{i}(\Sigma_{s}\beta_{1,i,s}\;\times \;PA{\;}^{+}{\;}_{t,i,s})\;+\;\Sigma_{i}(\Sigma_{s}\beta_{2,i,s}\;\times \;PA^{-}{}_{t,i,s})\;+\;\Sigma_{p}\beta_{3,t.p}\;\times \;ET_{t,p}\;+\;baseline_{t}$$

$$E\left(D_{t}\right)\;=\;\Sigma_{i}(\Sigma_{s}\beta_{1,i,s}\;\times \;PA{\;}^{+}{\;}_{t,i,s})\;+\;adjusted\text{-}baseline_{t}$$

where *PA* ^-^ is the indirect benign effect and *PA* ^+^  is the direct effect, and the adjusted baseline is: 

$$\Sigma_{i}(\Sigma_{s}\beta_{2,i,s}\;\times \;PA^{-}{}_{t,i,s})\;+\;\Sigma_{p}\beta_{3,t.p}\;\times \;ET_{t,p}\;+\;baseline_{t}$$

However, information on how to separate direct (*PA* ^+^ ) and indirect benign (*PA* ^-^) effects of a pathogen are not readily available. Nevertheless, an approximation may be achieved by post-estimation assigning weeks with an overall benign effect as a *PA* ^-^ week, and include the effect in the adjusted baseline. Based on Formula (1), the approach approximates *PA* ^+^ *_t,i,s_* with *PA_t,i,s_ × I*(*β_1,i,s_* × *PA_t,i,s_* ≥ 0) and *PA* ^-^_t,i,s_ with *PA_t,i,s_* × *I*(*β_1,i,s_* × *PA_t,i,s_* < 0), where *I*(boolian) is an index equal 1 if ‘boolian’ is true else 0.

$$\begin{array}{l} E\left(D_{t}\right)\;=\;\Sigma_{i}(\Sigma_{s}\;\beta_{1,i,s}\;\times \;PA_{t,i,s}\;\times \;I(\beta_{1,i,s}\;\times \;PA_{t,i,s}\;\geq \;0))\;+\;\Sigma_{i}(\Sigma_{s}\;\beta_{1,i,s}\;\times \;PA_{t,i,s}\;\times \;I(\beta_{1,i,s}\;\times \;PA_{t,i,s}\;<\;0))\\ \;\;\;\;\;\;\;\;\;\;\;\;\;\;\;\;\;\;\;+\;\Sigma_{p}\;\beta_{2,t.p}\;\times \;ET_{t,p}\;+\;baseline_{t} \end{array}$$

$$\;\;\;\;\;\;\;\;\;\;\;\;\;=\;\Sigma_{i}(\Sigma_{s}\beta_{1,i,s}\;\times \;PA_{t,i,s}\;\times \;I(\beta_{1,i,s}\;\times \;PA_{t,i,s}\;\geq \;0))\;+\;adjusted\text{-}baseline_{t}$$

This approach, separating weeks into direct and indirect benign weekly effects for each pathogen will lead to an improved estimation of pathogen-direct effects on number of deaths, but will probably still represent an underestimation.

We propose one statistical time series regression model and two estimation approaches. A crude approach, estimating number of deaths attributable to a pathogen had there been no excess temperatures or other pathogens circulating (base model), and an adjusted approach, estimating number of deaths attributable to a pathogen adjusted for excess temperatures and benign effect of other pathogens (adjusted baseline model).

More details are presented in the Supplement.

### Indicators used for the AttMOMO model

Estimates of infection rates for a given pathogen in the population are often not available. An alternative is to use indicators with an assumed proportionality to number of infected persons, i.e. showing the same pattern over time. Commonly used indicators are often based on a combination of clinical symptom surveillance and virological sampling. The former, reflecting pathogen circulation dynamics in the population, and the latter adjusting for a potential over-diagnosing. The Goldstein-index is a well-established example, where the infection rate is estimated as the rate of symptoms multiplied by the rate of positive tests [5,7].

Data on symptom surveillance in a population will often come from the sentinel network of general practitioners, weekly reporting number of patients with for example influenza-like illness (ILI). If primary sector symptom surveillance is not in place, then a secondary sector severe symptom surveillance, based on hospital-diagnoses, may be used.

### COVID-19 indicator

For COVID-19, a population-based symptom surveillance was not in place from the beginning of the pandemic. An alternative severe symptom surveillance would be based on persons having a clinical COVID-19 diagnosis (International Classification of Diseases (ICD)10: B342A, B972A, Z038PA1).

In Denmark, all hospital contacts and all sample results are available, i.e. covering the entire population. Hence, as COVID-19 indicator, we used the Goldstein severe COVID-19 like symptoms (*GSCLS*) indicator:

$$\begin{array}{l} GSCLS\;=\;\left(\text{persons with a COVID-19 diagnosis}/\text{100,000}\;\text{population}\right)\;\\ \;\;\;\;\;\;\;\;\;\;\;\;\;\;\;\;\;\times \;\left(\text{persons with a SARS-CoV-2 positive test}/\text{100,000}\;\text{population}\right) \end{array}$$

where one person was only counted once in a week.

### Influenza indicator

Unfortunately, because COVID-19 and influenza symptoms are alike, the existing surveillance of influenza in the sentinel networks of general practitioners was jeopardised in Denmark. Therefore, in line with what was used for COVID-19 symptom surveillance severe influenza and pneumonia like symptoms (SIPLS), could be defined as having a clinical diagnosis of influenza or pneumonia (ICD10: J09 to J18), and used in combination with confirmed cases in an alternative Goldstein index, the Goldstein severe influenza and pneumonia like symptoms (*GSIPLS*).

$$\begin{array}{l} GSIPLS\;=\;\left(\text{persons with an influenza or pneumonia diagnosis}\;/\;100,000\;\text{population}\right)\;\\ \;\;\;\;\;\;\;\;\;\;\;\;\;\;\;\;\;\;\;\times \;\left(\text{persons with an influenza positive test}\;\text{/}\;\text{100,000}\;\text{population}\right) \end{array}$$

where one person was only counted once in a week.

### Excess ambient temperatures

Overall Danish daily temperatures were calculated as the average over daily temperatures from all Danish weather stations, weighted by the populations in the five Danish regions. Thereafter, weekly mean, minimum and maximum temperatures were calculated for each week. Based on these, we estimated the expected weekly minimum and maximum temperatures (*ptmin*, *ptmax*), using general linear models with a yearly seasonal variation, a sine curve.

Weekly excess temperatures were defined as having a mean weekly temperature (*temp*) above the expected maximum temperature or below the expected minimum temperature. Continuous, nominal values were defined as how far the mean weekly temperature were below or above the expected minimum or maximum, respectively.

ET = (temp – ptmin) × I(temp < ptmin) + (temp – ptmax) × I(temp > ptmin)

where I(boolian) is an index equal 1 if ‘boolian’ is true else 0.

### Mortality attributable to COVID-19 in Denmark

All analyses and results were done by week, using the ISOweek 8601 standard (YYYY-WNN, defined as Monday through Sunday) [8].

#### Data sources

All data were extracted in 2020-W22.

##### Deaths and population

Individual registrations of number of Danish citizens and all-cause deaths were obtained from the Danish civil registry. Delay in registration of deaths were corrected using the EuroMOMO delay-adjustment algorithm [9].

##### COVID-19 and influenza indicators

All Danish influenza and SARS-CoV-2 samples and test results were retrieved from the Danish Microbiological Database (MiBa) [10]. Individual registrations of diagnoses were obtained from The National Patient Register.

##### Ambient temperatures

Data on daily temperatures registered at Danish weather stations were downloaded from the National Oceanic and Atmospheric Administration Online Climate Data Directory [11]. Country data are available as comma-separated values (csv)-files on request from the EuroMOMO project (euromomo@ssi.dk). 

#### Modelling

We estimated number of deaths and mortality attributable to COVID-19 and influenza from 2019-W40 to 2020-W20. For analyses of the COVID-19 pandemic, we also report weekly numbers from 2020-W09, i.e. the week with the first registrations of SARS-CoV-2 circulation in Denmark, to 2020-W20. We compared these to case fatalities as of date of deaths, i.e. number of deaths, where the deceased had a positive SARS-CoV-2 test within 30 days before death.

With relatively few excess deaths in Denmark, age group specific analyses will be unstable, therefore we limit our reporting to all ages only. However, as there are differences between age groups in mortality pattern over time as well as in the effects of influenza and COVID-19, we also report an age-stratified total, achieved by pooling over the age groups 0–14, 15–44, 45–64, 65–74, 75–84 and ≥ 85 years.

Experience from the EuroMOMO and FluMOMO models has shown that five preceding seasons will be sufficient to estimate a stable baseline with a linear trend to adjust for changes in the population. A longer period demands a more flexible trend. However, more flexible trends, such as splines, will be unstable at the ends, and will therefore not be suitable for the current focus on timely estimation of COVID-19 mortality in the most recent weeks. Therefore, five preceding seasons and the present study period were used i.e. from 2014-W27 to 2020-W20.

We included trend and half-yearly cycles if they contributed on a 5% significance level (p < 0.05), while yearly seasonality was included on a 10% level. Including 1 to 3-week lags for COVID-19, influenza and excess temperature removed residual autocorrelation.

All analyses were performed using R 4.0.2. R-scripts [12] and applied data are available on www.github.com/JensXII/AttMOMO.

### Ethical statement

This a register based study and an ethical approval was not needed.

## Results

**Figure 2:**
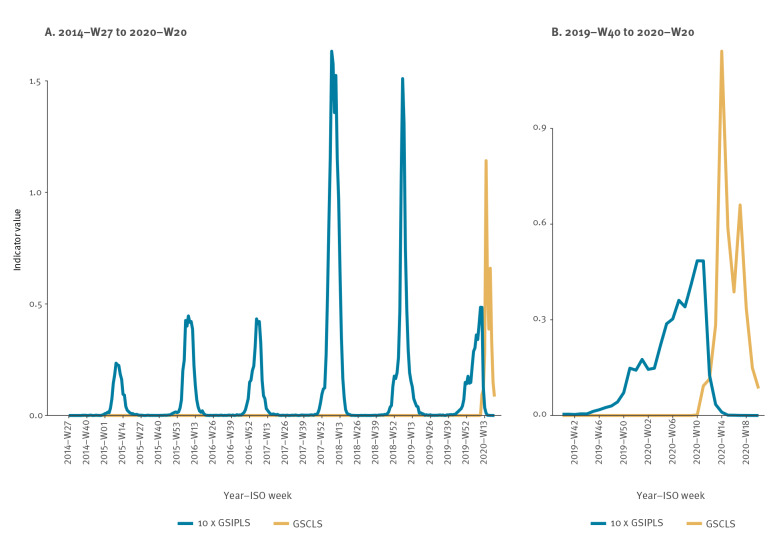
Distributions of severe COVID-19 (GSCLS) and severe influenza or pneumonia (GSIPLS)^a^ indicator values for all ages over (A) the whole estimation period and (B) 2019-W40–2020-W20, Denmark, 2014-W27–2020-W20

COVID-19: coronavirus disease; GSCLS: Goldstein severe COVID-19 like symptom indicator; GSIPLS: Golstein severe influenza or pneumonia like symptom indicator; ISO: International Organization for Standardization; W: week.

^a^ GSIPLS values are multiplied by 10, to make them visually comparable to GSCLS.

From 2014-W27 to 2019-W26, a median of 5,172 (range: 2,583–16,436) persons per season were positive for influenza and a median of 59,188 (range: 52,612–60,486) persons had an influenza or pneumonia diagnosis. From 2019-W40 to  2020-W20, a total of 7,602 persons had a positive influenza test and 44,534 an influenza or pneumonia diagnosis. For the COVID-19-diagnoses and SARS-CoV-2 tests, available in Denmark from mid-January 2020, 79,523 persons had a COVID-19 clinical diagnosis and 11,852 had a positive SARS-CoV-2 test from 2020-W09 to 2020-W20. Figure 2 shows the two indicators of influenza (*GSIPLS*) and COVID-19 (*GSCLS*). Indicators by age group are presented in the Supplement.

**Figure 3:**
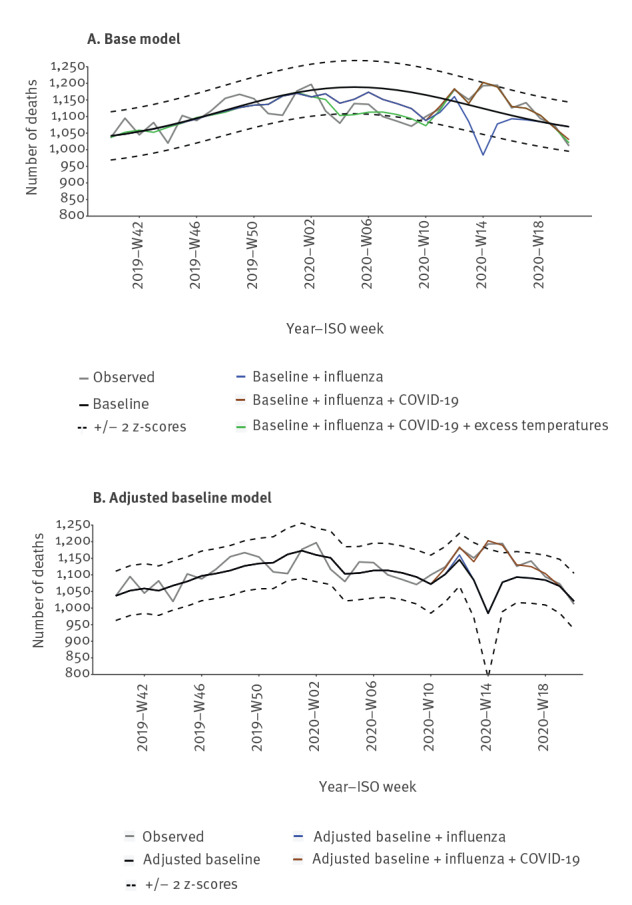
Estimated number of deaths for all ages according to (A) the base model and (B) the adjusted baseline model, Denmark, 2019-W40–2020-W20

COVID-19: coronavirus disease; ISO: International Organization for Standardization; W: week.

The regression fitted well (R^2^: 0.85) and there was no indication of heteroscedasticity or residual autocorrelation. Figure 3 shows the estimated number of deaths by the two estimation approaches (base model and adjusted baseline model) for the 2019/20 influenza season. Figures showing estimated number of deaths over the entire estimation period as well as by age groups are shown in the Supplement.

The number of influenza-attributable deaths, when including both benign indirect and direct effects and given there had been no excess temperatures or circulation of COVID-19 (base model) were estimated to 697 (95% confidence interval (CI): 505 to 889) fewer deaths than expected, pooled over all ages, which reflects a mild influenza season. However, when adjusting for benign indirect effects of excess temperatures and COVID-19 (adjusted baseline model) a pooled number of 119 (95% CI: 41 to 196) deaths were attributable to influenza, corresponding to an influenza-attributable mortality of 3.23 (95% CI: 1.12 to 5.35) per 100,000 person-years (Table 1).

**Table 1:** Estimated number of deaths and mortality attributable to influenza and COVID-19, Denmark, 2019-W40–2020-W20

Model	Population of 5.82 million	Influenza (GSIPLS)		COVID−19 (GSCLS)	
		Number of deaths (95% CI)	Number of deaths per 100,000 person−years (95% CI)	Number of deaths (95% CI)	Number of deaths per 100,000 person−years (95% CI)
Base	All ages	−646.1 (−898.3 to −393.9)	−17.62 (−24.50 to −10.74)	469.6 (230.9 to 708.3)	12.81 (6.30 to 19.32)
	Pooled over age groups	−697.1 (−889.3 to −504.9)	−19.09 (−24.26 to −13.77)	495.6 (313.3 to 678.0)	13.52 (8.54 to 18.49)
Adjusted baseline	All ages	14.7 (−61.0 to 90.4)	0.40 (−1.66 to 2.47)	519.1 (287.9 to 750.3)	14.16 (7.85 to 20.46)
	Pooled over age groups	118.6 (41.1 to 196.0)	3.23 (1.12 to 5.35)	594.1 (440.4 to 747.7)	16.20 (12.01 to 20.39)

CI: confidence interval; COVID-19: coronavirus disease; GSCLS: Goldstein severe COVID-19 like symptom indicator; GSIPLS: Goldstein severe influenza or pneumonia like symptom indicator; W: week.

For COVID-19, the estimated number of attributable deaths, including direct and indirect effects of COVID-19 (base model), pooled over age groups, was 496 (95% CI: 313 to 678) deaths, corresponding to a mortality of 13.52 (95% CI: 8.54 to 18.49) per 100,000 person-years. Adjusting for excess temperatures and benign indirect effects (adjusted baseline model), 594 (95% CI: 440 to 748) deaths were attributable to COVID-19, with a mortality of 16.20 (95% CI: 12.01 to 20.39) per 100,000 person-years (Table 1). 

**Figure 4:**
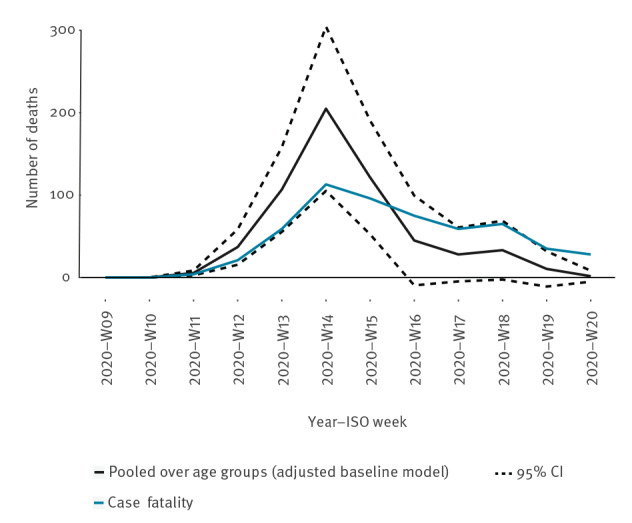
Weekly number of estimated deaths attributable to COVID-19 and case fatalities, pooled over age groups, Denmark, 2020-W09–2020-W20

COVID-19: coronavirus disease; ISO: International Organization for Standardization; W: week.

Weekly estimated number of deaths attributable to COVID-19, pooled over age groups, according to the adjusted baseline model together with weekly case fatalities from start of registered circulation in 2020-W09 to 2020-W20 are shown in Table 2 and Figure 4.

**Table 2:** Estimated number of deaths attributable to COVID-19 by week and 30 days case fatality, Denmark, 2020-W09–2020-W20

Model	Baseline number of deaths (95% CI)		Baseline adjusted number of deaths (95% CI)		30 days case−fatality (as of date of death)
ISO week	All ages	Pooled over age groups	All ages	Pooled over age groups	
2020−W09	0.01 (0.00 to 0.01)	0.00 (0.00 to 0.01)	0.01 (0.00 to 0.01)	0.00 (0.00 to 0.01)	0
2020−W10	0.35 (0.04 to 0.65)	0.07 (0.00 to 0.14)	0.35 (0.04 to 0.65)	0.08 (0.03 to 0.12)	0
2020−W11	18.4 (2.4 to 34.5)	5.3 (1.2 to 9.4)	18.4 (2.4 to 34.5)	5.6 (2.6 to 8.5)	4
2020−W12	23.3 (6.6 to 40.1)	35.7 (14.0 to 57.5)	23.3 (6.6 to 40.1)	37.1 (15.5 to 58.6)	21
2020−W13	55.1 (11.2 to 99.1)	105.8 (53.6 to 157.9)	55.1 (11.2 to 99.1)	106.8 (55.4 to 158.2)	59
2020−W14	219.0 (39.2 to 398.7)	199.8 (92.0 to 307.6)	219.0 (39.2 to 398.7)	204.8 (105.2 to 304.5)	113
2020−W15	111.4 (26.5 to 196.3)	113.9 (39.7 to 188.2)	111.4 (26.5 to 196.3)	121.5 (52.5 to 190.5)	96
2020−W16	37.1 (−34.4 to 108.5)	37.13 (−28.1 to 102.4)	37.1 (−34.4 to 108.5)	44.9 (−9.5 to 99.4)	75
2020−W17	34.9 (−32.5 to 102.3)	23.2 (0.38.1 to 84.6)	34.9 (−32.5 to 102.3)	28.0 (−4.7 to 60.7)	59
2020−W18	19.5 (−23.1 to 62.2)	26.2 (−16.9 to 69.4)	19.5 (−23.1 to 62.2)	33.2 (−2.46 to 68.9)	65
2020−W19	−10.4 (−48.3 to 27.5)	−5.7 (−43.3 to 32.0)	0.00 (0.00 to 0.00)	10.5 (−10.9 to 31.9)	35
2020−W20	−39.1 (0.84.9 to 6.8)	−45.9 (−86.0 to −5.8)	0.00 (0.00 to 0.00)	1.6 (−5.0 to 8.3)	28
Total	469.6 (230.9 to 708.3)	495.6 (313.3 to 678.0)	519.1 (287.8 to 750.3)	594.1 (440.4 to 747.7)	555

CI: confidence interval; COVID-19: coronavirus disease; ISO: International Organization for Standardization; W: week.

Estimated number of COVID-19-attributable deaths and case fatalities both peak in 2020-W14 with 205 and 113 deaths, respectively. Within the following 2 weeks, COVID-19-attributable number of deaths decline to below number of case fatalities, and thereafter they follow the same declining pattern.

## Discussion

The COVID-19 pandemic has caused substantial mortality in Europe. Indeed, a steep peak in all-cause excess mortality was observed in several European countries in March–April 2020, as demonstrated by the EuroMOMO network in a pooled analysis showing over 40,000 excess deaths in 2020-W14, although varying considerably across countries [4]. Denmark had the first confirmed COVID-19 case in 2020-W09, and the impact of the pandemic has been relatively minor, with a peak in all-cause excess deaths around 2020-W14, though within the expected normal range [13], and 555 30-day COVID-19 case fatalities up to 2020-W20 (Table 2). A large number of preventive measures were implemented in Denmark, as in other countries, with a full national lockdown from 2020-W12 until a gradual re-opening from 2020-W17. Initially, a containment strategy, with case tracing, was applied, but from 12 March 2020 (2020-W11), as the number of infected persons had increased dramatically, the strategy changed to mitigation. In this context, and also considering the testing capacity, testing-strategies for SARS-CoV-2 varied too.

An important indicator in following the COVID-19 pandemic and evaluating interventions is the population-based estimate of deaths attributable to COVID-19. As the influenza season was still ongoing when COVID-19 emerged and spread in Europe, there was a need to adapt the FluMOMO model to a more general model, allowing mortality estimations for multiple pathogens simultaneously. For this purpose, we developed a model for population-based assessment of attributable mortality to one or more pathogens simultaneously, the AttMOMO model.

During the COVID-19 pandemic, the implementation of non-pharmaceutical interventions to reduce the risk of infection by the targeted pathogen, SARS-CoV-2, may have also reduced the risk of other infections, e.g. influenza. In the Danish data, we observed that the influenza infection rate was reduced/not-existing during the lockdown of Denmark (Figure 2B), a phenomenon also observed in other Nordic countries [14]. Hence, circulation of one pathogen may both have potential direct malignant and indirect benign effects on mortality, due to associated preventive measures implemented to contain the target infection. Hence, the estimated attributable mortality will be a combined effect of the two. Separation of these effects was already a challenge in the one pathogen models used to estimate mortality associated to influenza [5], circumvented by repressing benign effects [15,16]. With several pathogens, the separation became unavoidable, as the indirect benign effect of one pathogen may obscure a harmful effect of another pathogen. We have not fully solved the challenge, but proposed an approximation, adjusting the expected mortality (baseline) to potential indirect benign effects on overall mortality due to implemented preventive measures as well as excess temperatures.

We present one model and two estimation approaches. One where attributable mortality to one pathogen is estimated assuming no circulation of other pathogens or any excess temperatures (base model), and another also adjusting for indirect benign effects from other pathogens as well as excess temperatures (adjusted baseline model). Estimated attributable mortality for one pathogen using the base model may be blurred by indirect benign effects of other pathogens, therefore, we recommend the adjusted baseline approach. We applied the model and both estimation approaches on data from the first wave, in spring 2020, of the COVID-19 pandemic in Denmark. Obviously, this can be extended to a longer time period and other countries.

In Denmark, as well as in most of Europe, the 2019/20 influenza season was mild, with an unusually low circulation of influenza, mainly A(H3N2) [17], and a relatively high vaccine effectiveness [18]. When adjusting for indirect benign effects during the season (adjusted baseline model), we estimated 119 excess deaths attributable to influenza, compared with a median of 474 over the seasons 2010/11 to 2016/17 [5].

Danish all-cause mortality attributable to COVID-19, during the first wave (2020-W09 to 2020-W20) of the pandemic, was estimated to 16.2 (95% CI: 12.0 to 20.4) per 100,000 person-years (Table 1) equivalent to 594 deaths. The COVID-19-attributable mortality varied strongly over the period (Table 2), peaking in 2020-W14 with 205 deaths (Table 2).

Within the first 5 weeks after COVID-19-attributable mortality had begun to increase in Denmark (2020-W11 to 2020-W15), the number of deaths attributable to COVID-19 was up to twice the 30-day case fatality, reflecting the undetected mortality burden in the population. From 2020-W16 and onward, the 30-day case fatality became higher than the population based attributable mortality, because even though a person had a SARS-CoV-2 infection within the last 30 days, some died from other causes. Furthermore, it is interesting to observe that case fatality and population-based attributable deaths follow the same pattern, which could indicate, that if a person dies due to COVID-19, it generally happens shortly after being infected.

The lockdown of Denmark and implementation of largescale preventive measures due to COVID-19, also reduced circulation of other infectious pathogens, leading to a decline in mortality from non-COVID-19 causes, especially in the first weeks of the lockdown (Figure 3). The lockdown would probably have saved 558 (95% CI: 299 to 816) lives from the start (2020-W12) to 2020-W20, if there had not been COVID-19 and the same preventive measures had been implemented.

The total number of infected persons in a population or infection rates is rarely, if ever, available. Therefore, we are restricted to use available indicators and assume these reflects number of infected persons in the population. We have used indicators based on secondary health sector contacts i.e. severe symptoms, thus missing persons with mild symptoms and asymptomatic cases. This may have led to an underestimation of number of deaths attributable to COVID-19 and influenza in the entire population. On the other hand, using standardised indicators will allow estimated attributable mortalities to be comparable across countries or regions. Further, the model assumes a constant relation between disease data (indicators) and mortality. However, for a novel disease like COVID-19, this constant relationship is likely to shift as clinicians become more adept at treatment and new drugs become available.

In summary, we have developed a statistical model, the AttMOMO model, and two estimation approaches for estimating attributable mortality from multiple pathogens simultaneously, and we have shown that they provide robust estimates of COVID-19 and influenza-attributable mortality, adjusted for seasonal mortality patterns and excess temperatures. Further, using standardised indicators for infection rates in the populations, the mortality estimates will be comparable between countries, and the model has the potential for timely monitoring and comparisons of mortality patterns across countries. The model and the two estimation approaches should be evaluated further on data from other countries or regions, including use of both standardised and locally available indicators. 

## Access to computing code and data

All analyses were done using R 4.0.2. R-scripts and used data are available on www.github.com/JensXII/AttMOMO.

## Supplementary Data

787000 Supplementary Material
