# Supplementary material for: Estimates of mortality attributable to COVID-19: a statistical model for monitoring COVID-19 and seasonal influenza, Denmark, spring 2020
Source: Euro Surveill. 2021 Feb 25;26(8):2001646. doi: 10.2807/1560-7917.ES.2021.26.8.2001646 (PMC7908066; doi:10.2807/1560-7917.ES.2021.26.8.2001646)

Supplement: Formulas, indicator- and model outcome figures

This supplementary material is hosted by *Eurosurveillance* as supporting information alongside the article “Estimates of mortality attributable to COVID-19: a statistical model for monitoring COVID-19 and seasonal influenza, Denmark, spring 2020”, on behalf of the authors, who remain responsible for the accuracy and appropriateness of the content. The same standards for ethics, copyright, attributions and permissions as for the article apply. *Eurosurveillance* does not edit supplements and the journal is not responsible for the maintenance of any links or email addresses provided therein.

Formulas

Notation

t	time in ISOweek
p	cold-winter, warm-winter, cold-summer, warm-summer
l	lag in ISOweek
D	Number of deaths
B	Baseline. Expected number of deaths, had there been no circulation of any of the pathogens (PAs) or excess temperatures (ET)
AB	Adjusted baseline. Expected number of deaths adjusted for indirect benign effects of circulation of other pathogens (PAs) and excess temperatures (ET)
PA_i	Indicator of pathogen PA_i
PA^+_i	Indicator of direct attributable effect of pathogen PA_i
PA^-_i	Indicator for indirect benign effect of pathogen PA_i
ET	Excess temperature
s	season, week 27 to week 26 the following year
Φ	Over dispersion parameter

Regression model

The full model, including lagged effects, can be written as:

$$E(D_t) = \Sigma_i (\Sigma_s \Sigma_l \beta_{1,i,s,l} * PA_{t,i,s,l}) + \Sigma_p \Sigma_l \beta_{2,t,p,l} * ET_{t,p,l} + B_t$$

where B_t, baseline, consists of an overall level (const), a linear trend, yearly and half-yearly seasonality:

$$B_t = \alpha_0 * const(=1) + \alpha_1 * t +$$
$$\alpha_2 * \sin(2\pi * (365.25/7) * t) + \alpha_3 * \cos(2\pi * (365.25/7) * t) +$$
$$\alpha_4 * \sin(4\pi * (365.25/7) * t) + \alpha_5 * \cos(4\pi * (365.25/7) * t)$$

A time series, multivariate, additive Poisson regression with overdispersion was used for estimation.

Baseline

Estimates of baseline $E(B_t)$ were achieved using the full model to predict the expected number of deaths over the same time period, had there been no circulating PAs or ET i.e. predicting from the full model with all PAs and ET set to 0 (the indicator values, not the regression coefficients),

thereby, using the full model covariances, i.e. correlations, between the baseline and the PAs and ET.

The baseline residual variance is then:

$$
\begin{aligned}
RV(B_t) &= V(observed_t - B_t) \\
&= V(observed_t) + V(B_t) \\
&= \Phi * E(observed_t) + V(B_t) \\
&= \Phi * E(B_t) + V(B_t)
\end{aligned}
$$

where Φ is over dispersion and $V(B_t)$ is the predicted regression variance.

Baseline reference intervals can be calculated by $E(B_t) \pm z * \sqrt{}RV(B_t)$.

PA and ET

A conditional approach was also used for estimating deaths attributable to a PA_j by predicting number of deaths had only that specific PA_j been circulating. Done by setting all baseline variables (const, t, sin, cos) to 0 as well as $PA_{j\neq i} = 0$ and ET = 0, thus estimating deaths attributable to PA_j.

The same procedure can be used for ET, with all PAs set to 0,

Adjusted baseline

A baseline adjusted for ET and indirect benign effect of the PAs, $E(AB_t)$, was achieved based on the above estimates of weekly effect of the PAs, setting weeks with a positive effect to zero i.e. including baseline B, ET and PAs in weeks with a combined indirect benign effect, and again using the full model to predict expected number of deaths with the observed ET, and benign effects of the PAs.

The residual variance for the adjusted baseline is:

$RV(AB_t) = V(observed_t - AB_t) = \Phi * E(AB_t) + V(AB_t)$

where Φ is over dispersion and $V(AB_t)$ is the predicted regression variance.

Baseline reference intervals can be calculated by $E(AB_t) \pm z * \sqrt{}RV(AB_t)$.

Adjusted PA and ET

A conditional approach was also used to estimate number of deaths attributable to a PA_j by predicting number of deaths had only that specific PA_j had direct i.e. positive, effect on number of deaths. In practice this is done by setting all baseline variables (const, t, sin, cos) as well as ET, PA^- and $PA^+_{j\neq i}$ to 0.

Cumulated numbers of attributable deaths

Mean cumulated number of attributable deaths to for example PA_i over w weeks:

$E(CPA_{i,w}) = \Sigma_w E(PA_{i,t=w})$

With independence between weeks i.e. no residual autocorrelation, the variance of the cumulated mean is:

$V(CPA_{i,w}) = V(\Sigma_w E(PA_{i,t=w})) = \Sigma_w V(PA_{i,t=w})$

Likewise for the estimated adjusted attributable deaths.

Indicators

Figure S1. Indicators, severe influenza or pneumonia (GSIPLS) and severe COVID-19 (GSCLS) by age group and total. The whole estimation period and the 2019/10 winter season.

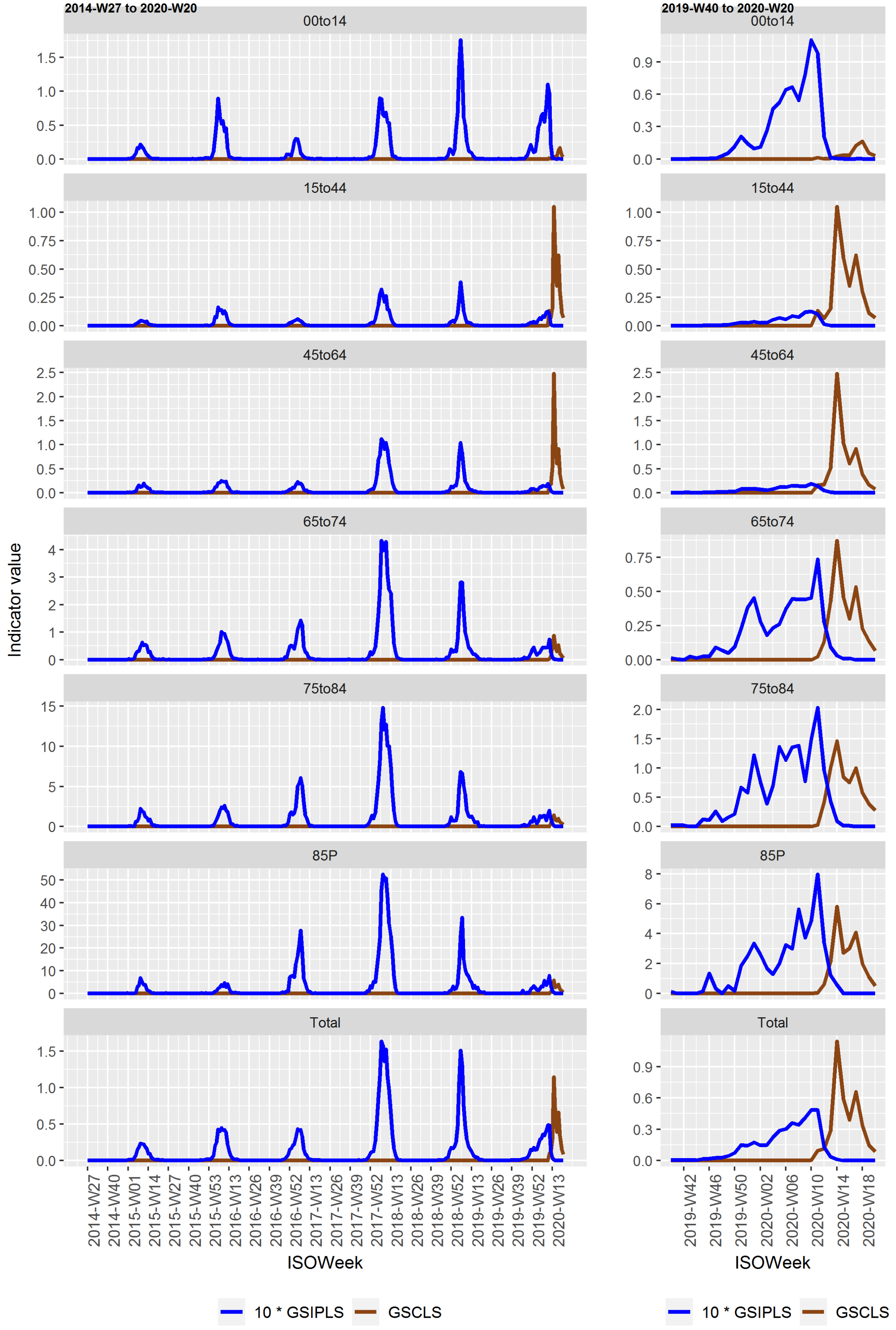

Model outcomes

Figure S2. Base model outcome 2014-W27 to 2020-W20 by age group and total. Influenza (GSIPLS) and COVID-19 (GSCLS)

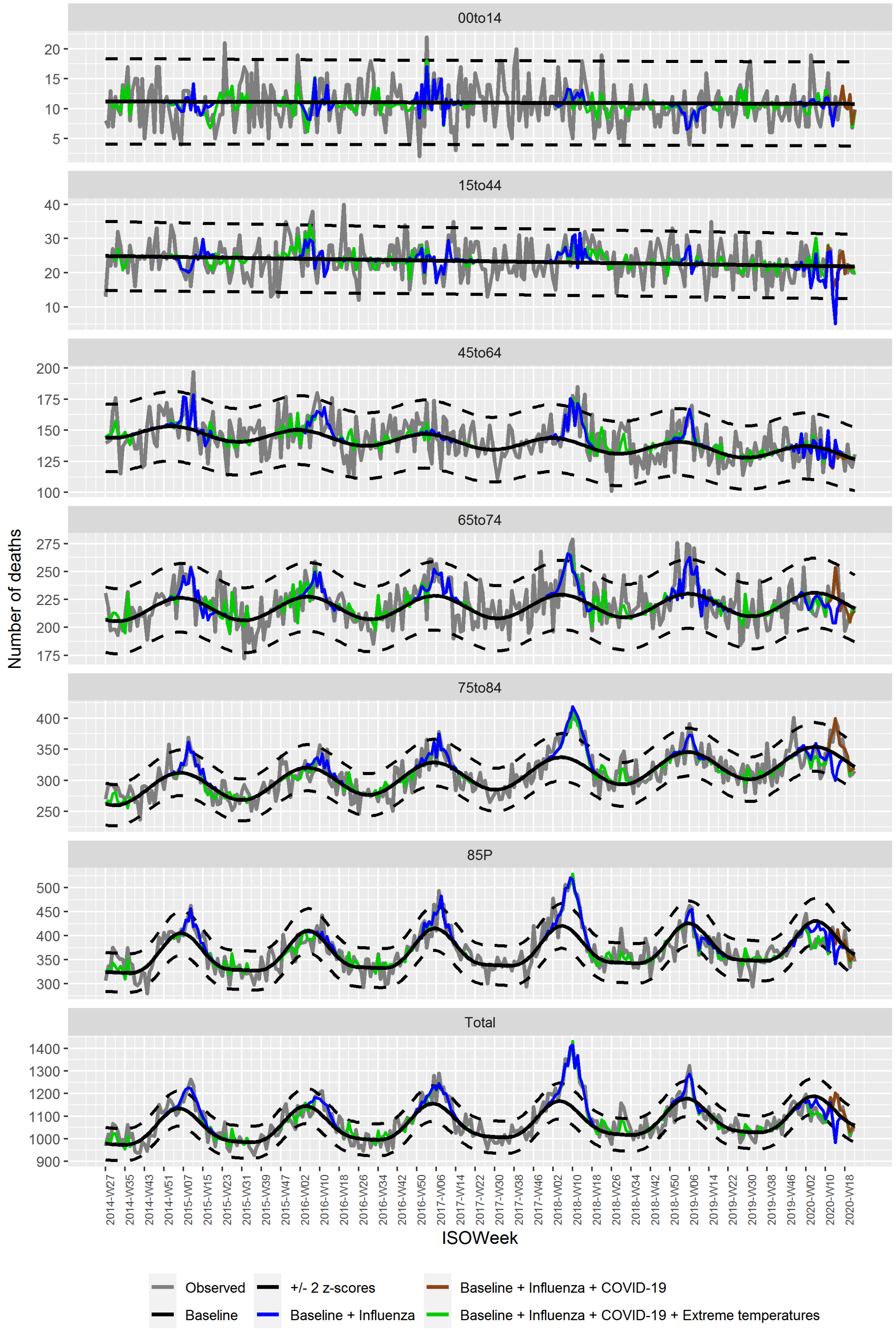

Figure S3. Adjusted baseline model outcome 2014-W27 to 2020-W20 by age group and total. Influenza (GSIPLS) and COVID-19 (GSCLS)

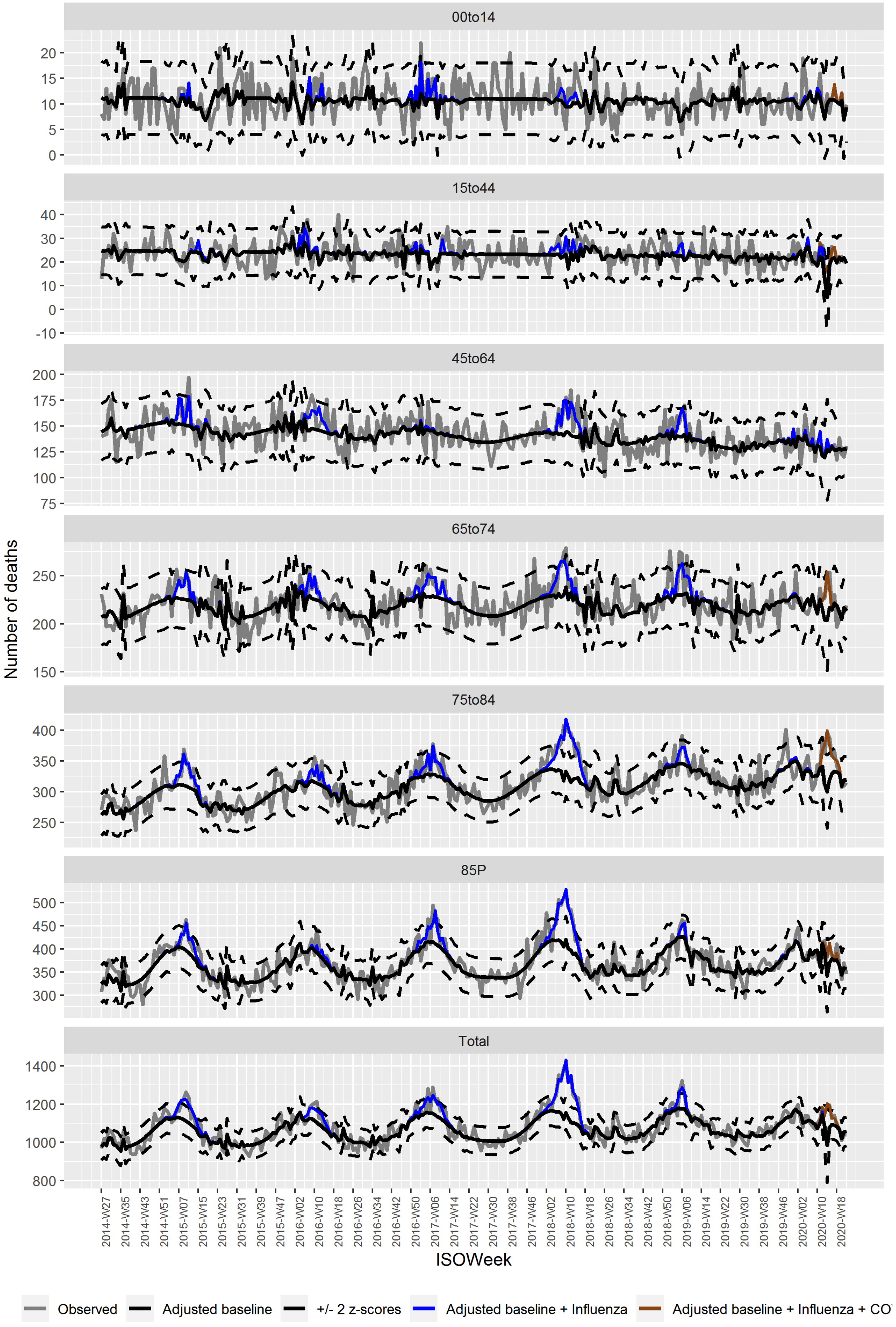

Supplement: Supplementary Material [file 2001646_Supplement.pdf]
